# Reviews

**Published:** 1882-05

**Authors:** 


### Reviews.

---

**A System of Surgery. Theoretical and Practical, in Treatises, by various Authors.** Edited by T. HOLMES, M. A., Cantab., Surgeon and Lecturer on Surgery, at St. George's Hospital. First American from second English edition. Thoroughly revised and much enlarged, by JOHN A. PACKARD, A. M., M. D., Surgeon to Episcopal and St. Joseph's Hospital, Philadelphia Assisted by a large corps of the most eminent American Surgeons. In three volumes, with many illustrations. Vol. III. Philadelphia: Henry C. Lea's Son & Co. 1882.

With this volume the work is completed, and the profession in possession of a reliable, complete and thoroughly scientific treatise on surgery. Vol. III treats of diseases of the respiratory organs, diseases of bones, joints and muscles, diseases of the nervous system, gunshot wounds, operative and minor surgery, and miscellaneous subjects; including diseases of the breast, diseases of the skin, parasites, surgical diseases of childhood, surgical diagnosis, and a large and interesting chapter on hospitals. As a book of references Holmes' Surgery is unrivalled, and as a text-book for the medical student it is not surpassed by any in the English language.

---

**A Study of the Tumors of the Bladder.** With original Contributions and Drawings. By ALEX. W. STEIN, M. D., Surgeon to Charity Hospital, N. Y., etc. New York: William Wood & Co., 27 Great Jones St. 1881.

The author with great diligence has gone through the literature, mostly of medical journals, and collected sixty cases of tumors of the bladder. With these as a foundation he has given us a clear and excellent monograph on the subject, so much more valuable, as our knowledge on this point is rather limited and confined to, what is found in the common surgical text-books. The chapters on symptomatology, diagnosis and treatment, are clear and instructive, and altogether the book is a valuable contribution to surgical literature.

---

**An Index of Surgery**; Being a concise Classification of the main Facts and Theories of Surgery for the Use of Senior Students, and others. By C. B. KEETLEY, F. R. C. S., Senior Assistant-Surgeon to the West London Hospital, etc. New York: Bermingham & Co, 1260 and 1262 Broadway. 1882.

The author's aim has been, as plainly indicated in the title, to furnish the student a work, embracing in a short compass all, the main facts and theories of surgery, by aid of which he may recapitulate the surgery shortly before his final examination. With this aim in view, the author has succeeded remarkably well, and produced a book, which will be of genuine advantage to the student.

---

**Percussion Outlines.** By. E. G. CUTLER, M. D., Assistant in Pathological Anatomy, Harvard Medical School, &c., and G. M. GARLAND, M. D., Assistant in Clinical Medicine, Harvard Medical School, &c. Boston: Houghton, Miflin & Co., 11 East 17th Street, New York. The Riverside Press, Cambridge. 1882. An octavo of 65 pages.

From the preface we learn that "this little book is intended to teach students the anatomical position of the thoracic and abdominal viscera in the living subject, and to portray such boundaries of these organs as are accessible to percussion. . . . With regard to the preparation of the book, we will add that it is essentially a condensed abstract of the German Literature upon this subject as contributed by Weil, Ferber, Luscha and Gerhardt. We have, however, repeatedly and carefully reviewed in our own practice and at the autopsy table the points which we present, and have convinced ourselves that they are correct." The book includes nine plates, as well as a number of diagrams. The subject all will admit is one the importance of which every physician daily feels at the bedside. Considering the data reliable, we recommend the work to our readers.

**Homœopathy: What is it?** By A. B. Palmer, M. D., L. L. D., Professor of Pathology and Practice of Medicine in the College of Medicine and Surgery in the University of Michigan. Second Edition 1881. Detroit, Mich: Geo. S. Davis, Medical Publisher.

We have many times been impressed with the remarkable want of information on the part of the profession generally, regarding the essentials of homœopathy. The introduction to this little pamphlet notices this fact in the following words: "Very many educated and even many scientific men have extremely vague ideas on the subject of medicine and the different medical schools. They have an indistinct, erroneous impression that there is some specific system of 'old school' medicine called Allopathy, of a similar exclusive character, but opposed to the 'new school' system of Homœopathy, and are without any definite, much less technical, knowledge of either. . . . They are ignorant of the fact that regular scientific medicine is no exclusive dogmatic system at all." That this is a very general fact is not to be denied. For this reason any work like the present tending to elucidate the theory and practice of Homœopathy should receive general examination. The author has given the subject much time and thought, and the result is a book which no physician should fail to read.

---

**The Diagnosis and Treatment of the Diseases of the Eye.** By Henry W. Williams, A. M., M. D. Boston: Houghton, Mifflin & Co.

Former editions of this work are so well known as to make an extended notice of this unnecessary. Yet as there are several points in which a marked improvement over previous issues is exhibited, it demands a few words setting forth anew its advantages for physicians in general and students in particular. The style is concise and clear, and the arrangement eminently practical, the author's aim evidently being to embody in as simple a manner possible, the result of his own personal observation together with the recorded experience of others.

The first chapter is devoted to the method of examining the eye, intelligibly detailing the process in all its aspects; a plan highly commending itself as a departure from the usual course, other ophthalmic works having the information regarding examinations usually scattered, and obliging the student to glean items, as best he can, on this important point. Different affections of the globe are treated in order in subsequent chapters, a special one being assigned to each anatomical part. It is to be regretted that the wood-cuts are not more numerous; a great advantage is gained when a subject is thoroughly illustrated, and an appreciation of this fact by the author would have supplemented well the clearness of his text. The general characteristics, however, of the book are such as to render it an excellent guide, and to those whose practice does not afford the same extended opportunities as Dr. Williams', or for students in this especial branch of surgery, it will be found exceedingly valuable.

---

**A Practical Treatise on Hernia.** By JOSEPH H. WARREN, M. D. Second and revised edition. Fully illustrated. Boston: James R. Osgood & Co. 1882.

This work is a second edtion of the author's former work, "Hernia: strangulated and reducible," etc., in which he advocated the Heatonian method by injection for the radical cure of reducible hernia, and modified the fluid and the instruments used. The author has added six new chapters on the causation of hernia, recent operations for hernia, artificial anus, and wounds of the intestines, hydrocele and varicocele, observations on hernia and résumé and clinical reports, and left out the lengthy and tedious introduction of the former edition. As a record and advocate of the Heatonian method, the book is of interest, but when the author flatters himself with the belief, that he has written "a thoroughly comprehensive and practical text-book on hernia," we doubt, that this opinion will be endorsed by the profession. The book is a curious mixture of important and unimportant facts, which sometimes have nothing whatever to do with the subject, and is too broad and lengthy to be comprehensive and practical.

